# Toxicity Profile of Procarbazine Lomustine and Vincristine Chemotherapy in Low-Grade Glioma - Retrospective Review

**DOI:** 10.7759/cureus.11070

**Published:** 2020-10-20

**Authors:** Nabia Irfan, Eileen Samuel, Fajar Rafi Ranjha, Asmara Waheed, Muhammad Abu Bakar, Sadaf Usman, Sumera Butt, Asma Rashid, Irfan Yousaf

**Affiliations:** 1 Clinical and Radiation Oncology, Shaukat Khanum Memorial Cancer Hospital and Research Center, Lahore, PAK; 2 Biostatistics and Epidemiology, Shaukat Khanum Memorial Cancer Hospital and Research Center, Lahore, PAK; 3 Clinical Oncology, Colchester General Hospital, Colchester, GBR; 4 Surgery, Shaukat Khanum Memorial Cancer Hospital and Research Center, Lahore, PAK

**Keywords:** pcv, procarbazine lomustine and vincristine, toxicity, gliomas, lgg, low grade glioma, alt (alanine aminotransferase), sd (standard deviation), ecog (eastern cooperative oncology group), ast (aspartate aminotransferase)

## Abstract

Background

The role of Procarbazine Lomustine and Vincristine (PCV) chemotherapy is already established in terms of improving survival in low-grade glioma (LGG). This improved survival has led to the increasing administration of PCV to LGG patients over the past years. However, like other chemotherapies, serious hematological and non-hematological toxicities may occur. The purpose of this study was to evaluate the toxicity profile of PCV and its clinical relevance in our practice.

Materials and Methods

We reviewed 63 patients of LGG retrospectively who received chemotherapy PCV between January 2015 and January 2018 at Shaukat Khanum Memorial Cancer Hospital & Research Centre, Lahore.

Results

Significant hematological toxicity as grade 3 anemia, thrombocytopenia, and neutropenia occurred in 19%, 27%, and 46% respectively with PCV. Other toxicities such as neurotoxicity, vomiting and derangement of liver enzymes occurred in 3.2%, 19%, and 19% respectively. Patients who were on concurrent anticonvulsants had no increase in PCV toxicity. Survival was not impacted by hematological toxicities up to grade 3.

Conclusion

PCV chemotherapy is associated with major hematological, hepatic, and clinical toxicities (vomiting, constipation, and neuropathy). Hematological toxicities influenced the course of treatment in terms of delays and interruptions.

## Introduction

Low-grade glioma (LGG) is somewhat a rare disease entity, which constitutes about 15% of all primary brain tumors, occurring commonly in young adults [1]. Several studies have examined tumor-, patient-, and treatment-related prognostic factors to identify low-risk and high-risk groups in this population of patients. Tumor-related factors included tumor histology, tumor grade, the maximum diameter of the tumor, tumor location, contrast enhancement, and molecular markers. Astrocytoma histology, tumors >5cm, and the ones crossing midline were observed to do worse than their counterparts. Patient-related factors included patient age, performance status, neurologic deficits or mental changes, the presentation with seizure activity, and pre-diagnostic symptom duration. Old patients with poor performance status who presented with neurological deficits before surgery rather than seizures were held poor prognostic factors. Treatment-related factors were not definitively identified since most of the patients had surgery and radiotherapy. However, initial corticosteroid dependency was bad [2-5]. IDH1 codon 132 mutation and 1p/19q co-deletion constitutes an important genetic and molecular profile [6-7]. The impact of these mutations on the clinical course of glioma has led to a change in WHO classification of low-grade gliomas in 2007 [8].

A change in treatment paradigm resulted when prospective randomized trials revealed significant progression-free and overall survival, especially in select LGG patients. Maximum response in terms of tumor regression with survival benefit was found to be in one's who were older than 40 years or less than 40 with subtotal resection or biopsy and ones with 1p/19q co-deletion [9-10]. The approach of the addition of chemotherapy to surgery and radiotherapy was adopted at our center following the availability of data from randomized and prospective clinical trials which showed improvement in both progression-free survival and overall survival in LGG.

However, PCV is known for major toxicities which need to be taken into consideration. The most vexing toxicity was myelosuppression. Grade 3 or 4 hematological toxicity was experienced by almost one-third of patients. However, very few patients required red-cell transfusions or platelet transfusion, and rarely a case of neutropenia was reported. The most common side effects were grade 1 or 2 fatigue, anorexia, nausea, and vomiting. Procarbazine has been implicated as a cause of granulomatous hepatitis. CCNU‐induced liver abnormalities have been reported in up to one-fourth of patients and are usually mild and revert to normal in no time. Vincristine has seldom been implicated as a hepatotoxin. Vincristine induced a glove‐and‐stocking distribution of sensory neuropathy in 35% to 45% of patients. Motor and autonomic neuropathies were also significant with vinca alkaloids. Less commonly, ocular palsies and vocal cord paralysis developed with it with rare acute motor neuropathy, similar to Guillain‐Barré syndrome. The neurological symptomology was strongly dose‐dependent, largely reversible, and persistent neuropathy was found to have an important impact on patients' lives. One-third of patients had delayed treatment due to treatment-related toxicities. Modification of the dosage in the face of hepatic or renal dysfunction is probably advisable [9-15].

The primary intention to carry out our study was to acquire knowledge of the toxicity profile of PCV in our patient population, whereas, secondarily it was investigated if toxicity had an impact on patient survival.

## Materials and methods

Patient cohort

This study included total patients (n=63) with LGG who received chemotherapy PCV between January 2015 and January 2018 at Shaukat Khanum Memorial Cancer Hospital & Research Centre, Lahore. All patients had a diagnosis of Grade 2 either oligodendroglioma, oligoastrocytoma, or astrocytoma. The patients received PCV at some point either in the adjuvant setting or at progression. All patient's electronic record was reviewed retrospectively. Ethical approval was granted by the Institutional Review Board, Shaukat Khanum Memorial Cancer Hospital & Research Centre Lahore, Pakistan.

PCV Cycles

PCV chemotherapy consisted of 6 cycles (Procarbazine at a dose of 60mg/m^2^ orally per day on days 8-21 of each cycle, Lomustine at a dose of 110mg/m^2^ orally on day 1 of each cycle, and vincristine at a dose of 1.4mg/m^2^ (maximum dose of 2mg) administered intravenously on days 8 and 29 of each cycle). The cycle length was 6 weeks. The interval between each cycle was 6 weeks to quantify for treatment delays. We documented the number of cycles received without 1 or more drugs of PCV (if procarbazine, vincristine, and or lomustine were omitted). It was possible to document the number of cycles with dose reductions.

Medication

All anticonvulsants (Levetiracetam, Valproic acid and Carbamazepine) were documented.

Toxicity

Toxicities reviewed were hematological, hepatic, renal, and clinical (Neurological / Nausea/vomiting / Constipation). PCV toxicity period was defined from the first day of chemotherapy to 6 weeks from day 1 of the last cycle. For laboratory toxicity, the presence or absence of abnormal value was determined and toxicity was graded on Common Toxicity Criteria for Adverse Events version 4.0. All patients had CBC (complete blood count) and biochemistry (ALT, AST, Alkaline phosphatase, bilirubin, and creatinine) before Day 1, 8, and 29 of each cycle. The presence and absence of neurotoxicity, Constipation, Nausea, and or Vomiting was also collected using clinical note of doctors in the patient’s electronic file (HIS system)

Statistical Analysis

Kaplan-Meier survival curves were used to determine the impact of toxicity on survival. Comparisons between the various toxicities experienced and treatment delays or interruptions were made using a Chi-square test with a significant threshold of p-value at 0.05. All the analysis were conducted using the Statistical Package for the Social Science System (SPSS 20).

## Results

Patient and Tumour Characteristics

Patients in this study had a mean age of 38.9 years (range 20 to 59) and 69.9 % (n=44) of them are males. Neurological symptoms at diagnosis were found in 57.1 % (n=36). In 79.4 % (n=50) of our patients, epilepsy was documented as a symptoms present at diagnosis. The majority of patients had an Eastern Cooperative Oncology Group (ECOG) status of 0 or 1 (Table 1).

**Table 1:** Patient and tumor characteristics who received PCV between 1 January 2015 and 15 January 2018 LGG = low-grade gliomas; PCV = procarbazine, lomustine, and vincristine; SD = standard deviation; ECOG = Eastern Cooperative Oncology Group.

Patient and tumor characteristics	Patients (%)
Gender	
Female (%)	19 (30.2)
Male (%)	44 (69.9)
Age at diagnosis	
Mean±SD	38.9 ± 9.1
Presence of neurological symptoms at diagnosis	
Yes (%)	36 (57.1)
No (%)	27 (42.9)
Epilepsy at diagnosis	
Yes (%)	50 (79.4)
No (%)	13 (20.6)
ecog, mean±SD	
0 (%)	17 (27)
1 (%)	38 (60.3)
2 (%)	8 (12.7)
Oligodendroglioma	
Grade 2 (%)	16 (25.4)
Oligoastrocytoma	
Grade 2 (%)	2 (3.2)
Astrocytoma	
Grade 2 (%)	38 (60.3)
Diffuse Glioma	
Grade 2 (%)	7 (11.1)
1p19q	
Codeleted (%)	19 (30.2)
Non codeleted (%)	14 (22.2)
Unknown (%)	30 (47.6)
IDH1	
Mutated (%)	40 (63.5)
Wild type (%)	9 (14.3)
Unknown (%)	14 (22.2)
ATRX	
Loss (%)	25 (39.7)
Retained (%)	15 (23.8)
Unknown (%)	23 (36.5)

Tumor characteristics are also presented in Table 1. Oligodandroglioma accounted for 25.4% (n=16) of diagnosis while oligoastrocytoma and diffuse glioma was diagnosed in 3.2 %( n=2) and 11.1 %( n=7) of the patients respectively. Lastly astrocytoma was identified in 60.3% (n=38) of the individuals included in this study. The 1p19q co-deletion was present in 30.2% (n=19) and non co deletion was in 22.2% (n=14) but not available for 47.6% (n=30). Isocitrate dehydrogenase 1 mutation was present in 63.5% (n=40) and IDH wild type was in 14.3% (n=9) but unknown for 22.2% (n=14). ATRX loss was present in 39.7% (n=25) and ATRX retained 23.8% (n=15) but unknown for 36.5% (n=23). Majority of patients received PCV in adjuvant setting 96.8% (n=61) and 3.2% (n=2) received PCV on recurrence.

Toxicity

The main purpose of this study is to describe PCV toxicity (Table 2). A total of 76.2% experienced hematological toxicity. Grade 3 neutropenia and thrombocytopenia occurred in 46% (n=29) and 27% (n=17) of patients respectively. Grade 3 anemia occurred in 19% (n=12).

Liver toxicity was observed in 19% (n=12) with rise in aminotransferase (AST) in 15.9% (n=10) and rise in ALT in 9.5% (n=6) of the patients. Only 1.6% (n=1) had grade 3 rise in ALT.

A total of 30.2% (n=19) of patients had clinical toxicities while 3.2% (2) experienced neurotoxicity (Table II). Concerning gastrointestinal toxicity, 9.5 % (n=6) had constipation, 19% (n=12) had vomiting and 14.3 % (n=9) had nausea despite anti-emetic protocol during PCV administration.

**Table 2:** Toxicity Profile of PCV AST = aspartate aminotransferase; ALT = alanine aminotransferase.

Toxicity	Patients (%)	
	All grade	Grade 3–4
Hematological		
Anemia (%)	22 (34.9)	12 (19)
Neutropenia (%)	44 (69.8)	29 (46)
Thrombocytopenia (%)	30 (47.6)	17 (27)
Hepatic		
High AST (%)	10 (15.9)	0
High ALT (%)	6(9.5)	1(1.6)
High alkaline phosphatase (%)	7 (11.1)	0
High bilirubin (%)	2 (3.2)	0
Renal		
High creatinine (%)	1 (1.6)	1 (1.6)
Clinical		
Allergic reaction (%)	1 (1.6)	
Neurotoxicity (%)	2 (3.2)	
Constipation (%)	6 (9.5)	
Nausea (%)	9 (14.3)	
Vomit (%)	12 (19)	

Impact of hematological toxicity on survival

In our study, hematological toxicity was not related to mortality (Table 3).

**Table 3:** Impact of Hematological toxicity on survival mOS = Median overall survival; 3y OS = overall survival at 3 years; NR = not reported.

Survival classified by hematological toxicity	Toxicity experienced/Death	No toxicity/Death	P-Value
≥ grade 3 anemia	N=12/0	N=50/5	0.30
mOS (mo)	NR	NR	
3y OS, (%)	100	87	
≥ grade 3 neutropenia	N=29/2	N=33/3	0.65
mOS (mo)	NR	NR	
3y OS, (%)	90	87	
≥ grade 3 thrombocytopenia	N=17/1	N=45/4	0.80
mOS (mo)	NR	NR	
3y OS, (%)	94	88	

Impact of medications on toxicity

Our study did not reveal any correlation of anticonvulsants on the toxicity profile.

Impact of Toxicity on Treatment Course

Treatment delays occurred in 55.6% (n=35) of patients because of PCV toxicity (Table 4). A total of 38.1 % (n=24) of patients needed a dose reduction to pursue chemotherapy. Indeed, hematological toxicity influenced the course of treatment as patients who manifested abnormal platelet levels had significantly more delayed cycles( x2=28.411, df =2, p=0.000) than those who did not. The same correlation was found when comparing patients who experienced neutropenia and anemia with those who didn’t experience these (x2=14.023, df=2, p=0.001) and (x2=8.993, df = 2, p=0.01) respectively. The same correlation was not found when comparing patients who experienced neurotoxicity with those free of neurological symptoms (x2=0.301, df = 2, p=0.860). Finally, 33.3% (n=21) of patients had at least 1 incomplete cycle as 1 or more medication was stopped during treatment due to toxicity.

**Table 4:** Impact of PCV toxicity on the treatment course

Impact on treatment	Patients (%)
Delay of at least 1 cycle (%)	8(12.7)
Dose reduction (%)	24 (38.1)
Dose Reduction by Chemotherapy Agents	
CCNU (%)	15 (23.8)
Vincristine (%)	11 (17.4)
Procarbazine (%)	15 (23.8)
Complete discontinuation (%)	21 (33.3)

## Discussion

This paper intends to evaluate the toxicity of PCV in randomly selected patients who are diagnosed with LGG. The patients for this study have received chemotherapy during the time span of 1st January 2015 to 15 January 2018.

The results of our study are in line with the previous literature regarding the hematological toxicity of PCV [11]. Grade 3 hematological toxicity was observed in more than one-third of our randomly selected patients. This adverse event occurred in 28.1% of patients as suggested by the studies conducted by Kim et al [14]. Our study shows that 27% of patients have developed grade 3 thrombocytopenia which requires transfusion while the patients are receiving chemotherapy. Grade 3 neutropenia was also observed in many of our patients. Neutropenia can negatively impact the health and well being of patients and can lead to febrile neutropenia [16]. There was no toxic death at our center. However, hematological toxicity calls for requisite attention and must be addressed with utmost concern. 

The results of our study for hepatic toxicity are also in line with the previously conducted studies. It was demonstrated by King et al that procarbazine is associated with hepatic toxicity as hepatic enzymes are formed during its conversion. The elevation of aminotransferases was found to be associated with Lomustine. The modifications for the procarbazine dose were also suggested by King et al in case of hepatic toxicity [12]. We have meticulously observed our patients by keeping in view the above-mentioned things and determining the necessity for modification or omission of procarbazine. Our study shows that 7.9% of our patients have experienced grade 1-1 ALT toxicity and 15.8% of patients in our sample have experienced grade 1-2 AST toxicity. 

Scientific literature shows that the renal function of 10% of patients is impacted by PCV [11]. Our study shows that 1.6 % of patients had elevated levels of creatinine which can cause the reduction of Procarbazine/ Lomustine. The biochemistry is significantly impacted by the chemotherapy and patients on PCV should be closely monitored. 

Considering the clinically significant adverse events, neurotoxicity is a known adverse effect of vincristine. About 2% of the patients were reported to have polyneuropathy in the studies conducted by Van den Bent et al [17]. Our study shows that 3.2% of patients have experienced neurotoxicity which is in accordance with the results of the previous study. Our course of treatment was impacted due to clinical toxicity. 

Nausea, vomiting & constipation are the common side effects associated with the digestive tract. Our study shows that 14.3%, 19% & 9.5% of patients have suffered from nausea, vomiting & constipation respectively despite the fact that antiemetic protocol was followed for all our patients.

Chemotherapy is impacted by the adverse clinical events and can substantially retard the well-being of patients. It is, therefore, pertinent to recognize the importance of these adverse events. Patients tend to delay and in rare circumstances, discontinue PCV due to these adverse events even when they do not constitute life-threatening conditions.

The usage and administration of PCV have increased gigantically in the last decade. The increase can be attributed to the efficacy of PCV as demonstrated by recent studies and the improved outcomes in LGG when coupled with radiotherapy [10]. The incidence of toxicity must be examined by considering the impact of the line of treatment. It should also be determined whether patients have experienced more toxicity in adjuvant settings than those who have had experienced only during progression. 

PCV administration has many concerns due to the above-mentioned substantive toxicities. Previous studies show that 30% of the patients had to discontinue their chemotherapy on account of severe toxicities [9]. Our study shows that about 33.3% of our patients had to discontinue one or more medication of tri-therapy during our studies. A total of 12.7% of our patients have delayed at least 1 cycle and 42.9% of our patients have numerous sporadic interruptions due to the toxicity. Moreover, the dosage for 38.1% of patients was reduced due to the negative impact of toxicity. 

One of the advantages of our study was the relatively large sample size of our randomly selected patients. The random selection of patients and the compiled database can benefit other researchers and academics. Like other scientific work and previously conducted studies, our study too has its limitations. Certain elements of our research were conducted retrospectively and consequently, the subjective nature of our study limits the potential conclusions in different contexts and cultural settings. The duration of our study is 3 years whereas, a longitudinal study for measuring the toxicity of PCV with other parameters will be helpful. Structured follow-ups and recurrent blood sampling at regular intervals can also help us understand the toxicity of PCV. Other parameters and data such as quality of life could also help the researchers to find the toxicity of PCV in detail. 

## Conclusions

Our study investigated the toxicity profile associated with PCV with apparent manifestations of hematological, hepatic, renal, and clinical side effects. Concurrent anticonvulsant therapy does not appear to worsen toxicity. In view of the well-established role of PCV in LGG in terms of improvement in survival, a physician must proceed with caution especially in old patients with comorbidities before administering this regimen.
